# Factors predicting parent engagement in a family-based childhood obesity prevention and control program

**DOI:** 10.1186/s12889-023-15359-7

**Published:** 2023-03-08

**Authors:** Emily A. Schmied, Hala Madanat, Emmeline Chuang, Jamie Moody, Leticia Ibarra, Griselda Cervantes, David Strong, Kerri Boutelle, Guadalupe X. Ayala

**Affiliations:** 1 School of Public Health, San Diego State University, San Diego, 92182 USA; 2 Division of Research and Innovation, San Diego State University, San Diego, 92182 USA; 3 School of Social Welfare, University of California Berkeley, Berkeley, 94720 USA; 4 San Diego State University Research Foundation, San Diego, 92182 USA; 5 Department of Family Medicine and Public Health, University of California San Diego, La Jolla, San Diego, 92093 USA; 6 Department of Pediatrics, University of California San Diego, San Diego, 92161 USA; 7 Institute for Behavioral and Community Health, San Diego State University Research Foundation, San Diego, USA

**Keywords:** Childhood obesity, Parent engagement, Family-based interventions, Health promotion, Health education, Rural health, Hispanic/Latino health

## Abstract

**Background:**

Family-based interventions are efficacious at preventing and controlling childhood overweight and obesity; however, implementation is often hindered by low parent engagement. The purpose of this study was to evaluate predictors of parent engagement in a family-based childhood obesity prevention and control intervention.

**Methods:**

Predictors were assessed in a clinic-based community health worker (CHW)-led Family Wellness Program consisting of in-person educational workshops attended by parents and children. This program was part of a larger effort known as the Childhood Obesity Research Demonstration projects. Participants included 128 adult caretakers of children ages 2–11 (98% female). Predictors of parent engagement (e.g., anthropometric, sociodemographic, psychosocial variables) were assessed prior to the intervention. Attendance at intervention activities was recorded by the CHW. Zero-inflated Poisson regression was used to determine predictors of non-attendance and degree of attendance.

**Results:**

Parents’ lower readiness to make behavioral and parenting changes related to their child’s health was the sole predictor of non-attendance at planned intervention activities in adjusted models (OR = 0.41, *p* < .05). Higher levels of family functioning predicted degree of attendance (RR = 1.25, *p* < .01).

**Conclusions:**

To improve engagement in family-based childhood obesity prevention interventions, researchers should consider assessing and tailoring intervention strategies to align with the family’s readiness to change and promote family functioning.

**Trial registration:**

NCT02197390, 22/07/2014.

## Introduction

The effects of childhood obesity are serious and enduring [1, 2], and there is a critical need for effective prevention and control programs. Family-based interventions that target parents as agents of change are efficacious at preventing and controlling childhood overweight and obesity [3–6]. However, the implementation of these programs, and ultimately their efficacy and effectiveness, may be hindered by low parent engagement. For instance, researchers often report parent attendance at less than two thirds of program activities [7], and a 2014 review of 23 family-based child obesity interventions found a mean attrition rate of 41% [8]. Of great concern is the effect of parent engagement on whether intended program outcomes are achieved; research shows a direct relationship between parent engagement in child obesity prevention and control and child BMI and weight- related behaviors [9, 10].

To improve parent engagement in family-based childhood obesity prevention and control programs, it is crucial to determine factors that affect it. Despite a wealth of previous family-based programs [3, 11] there is a lack of consensus in the literature regarding predictors of parent engagement, as few studies have conducted prospective examinations, and fewer have tested theoretically-informed models [12]. What evidence is available suggests parent engagement may be affected by a variety of factors, ranging from sociodemographic (e.g., income level, parent marital status) to psychosocial (e.g., parent or child psychological health status; self-efficacy, family functioning) [12–16].

Engagement in family-based programs require, by definition, time and family support to participate. Parents frequently report that logistical challenges, including scheduling conflicts, competing priorities, and transportation issues impede their participation [13, 17, 18]. Further, single parent-households are often more likely to attrite from family-based programs [14, 16] which may be due to lack of time or scheduling difficulties [18]. In addition to logistical challenges, family functioning may play a role in engagement [19–21]. One family-based pediatric obesity program including 155 4–7 year old children [7] reported that family functioning was inversely related to program completion even after controlling for sociodemographic factors such as parent marital status and income. Similarly, in another study of 56 adolescents aged 11–18, parents and adolescents who dropped out of a family-based lifestyle intervention for obesity were more likely to report not getting along with each other than those who completed it [17].

Other factors that may influence engagement pertain to parent motivation and expectations. Parents who exhibit high degrees of motivation to participate and readiness to make health-related behavioral changes are often more likely to engage in the program [22–24]. For instance, one study that examined reasons for attrition among 242 participants of a family-based child obesity treatment program found that parents with lower motivation to participate at the start of the program were more likely to end the participation early [20]. Moreover, Kelleher et al. [14] noted that parents who enrolled in a community-based lifestyle program reported greater levels of concern for their child’s health and wellbeing. In contrast, parents who did not believe their child was overweight/obesity, and/or in need of intervention, reported greater barriers to participating or were more likely to drop out [25, 26].

The study of parent engagement is further complicated by the involvement of another factor— the participating child. Previous research suggests parents with older children are less likely to engage in programs [8, 22, 27]. Several studies have also documented a relationship between child depression or stress and reduced family participation in program activities [17, 28]. For example, a study by Fagg and colleagues of children ages 7–13 participating in a family-based weight management program were more likely to drop out if reporting greater psychological distress [28]. Finally, evidence suggests that child’s baseline weight may inversely relate to parent participation, though results have been inconsistent and are largely drawn from clinical treatment studies [8, 22].

While previous research has identified several potential predictors of parent engagement, few comprehensive, theoretically driven, prospective examinations have been conducted, leaving a gap in understanding how to intervene. This study prospectively examined potential predictors of parent engagement in a family-based childhood obesity prevention and control intervention conducted in Imperial County, California. It is hypothesized that personal characteristics of the parent ( receipt of public financial or food assistance, older age, married, lower BMI) and child ( younger age, lower BMI), as well as psychosocial factors ( greater readiness to change, greater perceived relevance of the intervention, better family functioning, lower perceived participation barriers, and less frequent parent and child psychological symptoms) will be predictive of greater engagement. It is also hypothesized that the predictors of engagement may differ by child baseline BMI classification. The results of this study will be important to inform development of strategies to improve engagement.

## Methods

### Design

This study used a prospective, longitudinal design to examine predictors of parent engagement in one component of the Imperial County, California, Childhood Obesity Research Demonstration study (CA-CORD). The objective of CA-CORD (conducted January 2012-June 2015), was to prevent and control childhood obesity by improving four weight-related behaviors: fruit and vegetable consumption, water consumption, physical activity, quality sleep. CA-CORD used a quasi-experimental pre/post-test design with three intervention arms and one control group, and implemented intervention strategies in five sectors: (1) healthcare, (2) early care and education centers, (3) schools, (4) community recreation organizations, and (5) restaurants. It was designed and implemented via a partnership between San Diego State University Research Foundation’s Institute for Behavioral and Community Health, *Clínicas de Salud Del Pueblo*, Inc., and the Imperial County Public Health Department. The full design and protocol of CA-CORD is described elsewhere [29]; it was registered as a clinical trial 22/07/2014 (Trial registration: NCT02197390).

The present study examined predictors of parent engagement in the Family Wellness Program, which was part of the CA-CORD healthcare sector intervention. The Family Wellness Program was included as part of an obesity care model implemented at *Clínicas de Salud Del Pueblo*, Inc., a large, federally-qualified health center. The program included a series of six healthy lifestyle workshops typically held weekly in small group settings (5–10 families per workshop). The workshops were led by trained community health workers (CHWs) and the content was rooted in health behavior change research and family systems theory [21, 30, 31]. Specifically, the evidence-based workshop curriculum was planned to promote health within the home by encouraging both parents and children to adopt healthy lifestyle behaviors by teaching them to navigate common challenges, such as social and structural barriers at home and in the community. For instance, parents received education on effective communication and parenting practices surrounding weight-related behaviors, including increasing parental capacity to set limits on certain behaviors, such as amount of screen time or sugary beverage consumption. Most workshop content was delivered to parents and children separately, though several joint activities were conducted. Families enrolled in the Family Wellness Program were also invited to attend a series of eight physical activity classes during the same six-week period as the lifestyle workshops. The physical activity classes taught families activities they could perform together at home. Parents received motivational interviewing phone calls at the start of the program and at quarterly intervals for the following year, to encourage attendance at workshops and classes, and the continued use of the new skills. Finally, parents received monthly educational newsletters. While the Family Wellness Program included many components, the outcome for the present study was attendance at the lifestyle workshops, as participation in the other components was either optional (i.e., physical activity classes) or passive (i.e., newsletters). All recruitment, informed consent, and measurement materials were approved by the SDSU Institutional Review Board and available in English and Spanish.

### Participants

CA-CORD participants included 1,186 children ages 2–11 and a primary caregiver. Families were recruited at school and community events and through the participating clinics. Exclusion criteria included: child BMI < 5th percentile; family plans to move outside of the county within 2.5 years; child is a foster child or has one of several health conditions that would hinder intervention participation. Due to the 2 × 2 design of CA-CORD, 50% of the families were assigned to the Family Wellness Program and were eligible to participate in the ancillary parent engagement study reported here (430 families, 526 children). CA-CORD parent participants enrolled in the Family Wellness Program were recruited for the ancillary study either in person or via regular mail prior to starting the intervention. In total, 128 of the 430 families (29.8%) agreed to participate in the present ancillary study. Group comparison testing (i.e., t-tests, Chi-square analyses) revealed no significant differences in demographic characteristics or BMI (all p > .05) between parents and children who participated in the present ancillary study and the larger CA-CORD program.

### Setting

Imperial County, CA lies along the US-Mexico border. A majority (85.0%) of the approximately 181,000 residents identify as Hispanic or Latino and 76.5% report speaking a language other than English in the home [32]. The region has poverty and childhood obesity rates that exceed state and national averages [32, 33].

### Measures

Data were collected via surveys and anthropometric assessments administered at baseline, and attendance records collected from parents and children throughout the program.

Parent engagement, the primary variable of interest in this study, was obtained using attendance records maintained by the CHWs during planned lifestyle workshops. Total number of workshops attended (0–6) by the participating parent was used in the analysis.

#### Parent and child sociodemographic characteristics

The following parent characteristics were assessed: age, gender, ethnicity (Hispanic, non- Hispanic white, other), marital status (married versus unmarried/separated/divorced), education (< 12th grade versus high school diploma/equivalent or higher). Child characteristics assessed included age and gender. Additionally, family socioeconomic status was assessed by collecting information about family enrollment in public food assistance programs, such as the Women, Infants, and Children program. Participants were coded as positive if they reported being enrolled in any public assistance program.

#### Perceived relevance and readiness to change

Parents’ perceived relevance of the intervention and their readiness to change their own health behaviors and parenting strategies related to their child’s weight and weight-related behaviors were assessed with two scales modified from the Parent Motivation Inventory [34]. Perceived relevance was assessed with 8 items (e.g., It is very important for the well-being of my child that they change their health behaviors) and readiness to change was assessed with 9 items (e.g., I am motivated to practice the techniques I will learn in CA-CORD at home with my child). Response options ranged from 1 (strongly disagree) to 5 (strongly agree) and mean scale scores were computed with higher scores indicating greater perceived relevance (α = 0.92) and readiness to make changes (α = 0.92), respectively.

#### Perceived barriers

Perceived barriers to participation were assessed with a 4-item scale based on the Barriers to Treatment Participation Scale [35] and other parent engagement research [17]. Parents were asked how much of a problem they thought four potential barriers may be for them to attend the Family Wellness Program: time, transportation, child’s willingness to participate, family support to participate. Response options ranged from 0 (not a problem) to 3 (serious problem). A mean scale score was computed, with a higher score indicating greater perceived barriers.

#### Family Functioning

Family functioning was measured with an abbreviated 3-item sub-scale from the third version of the Family Adaptation and Cohesion Scales [36]. The scale included items such as “My family members like to spend time with each other.” Response options ranged from 1 (strongly disagree) to 4 (strongly agree); item scores were averaged for analysis with higher scores indicating greater perceived family functioning (α = 0.94).

#### Psychological health symptoms (parent)

Symptoms of parent depression and anxiety were assessed with the 4-item version of the Patient Health Questionnaire [37]. Parents were asked how often in the past two weeks they felt bothered by various symptoms such as “feeling nervous, anxious or on edge.” Items were scored on a 4-point scale (1 = not at all to 4 = nearly every day), and scores were summed to compute a total score for analysis (α = 0.90).

#### Psychological Health Diagnoses (child)

Parents reported if their child had ever received a diagnosis from a physician for any of the following behavioral health disorders: depression, anxiety, attention deficit hyperactivity disorder. For analyses, responses were dichotomized into “none” and “1 or more.”

#### Parent perception of Child Weight

Parent perception of child weight was assessed with a figure rating scale [38]. Parents selected an image of a silhouette they believed corresponded to their child’s current body size; their selection was compared to their child’s actual BMI classification to determine if they over- or underestimated the child’s size. Responses were dichotomized for analysis (overestimated versus underestimated or correctly estimated).

#### Parent and child BMI

Trained staff measured parents and children’s height (cm) and weight (kg) to compute body mass index (BMI). For parents, BMI classification (< 25 healthy weight versus ≥ 25 overweight or obese) is reported for ease of interpretation, and raw continuous BMI scores were used for analyses ([kg]/ height[m]^2^). Similarly, for children, BMI percentage are reported for ease of interpretation, and BMI z-scores were used in regression analyses.

### Analysis

Descriptive statistics were computed to assess distribution of all study variables. Normality tests revealed the outcome (i.e., number of workshops attended) was not normally distributed (Shapiro-Wilk p < .05), indicating Poisson regression may be best-suited to examine predictors of engagement. To determine the most appropriate model, the fit of four regression models were compared: Poisson, negative binomial, zero-inflated Poisson, and zero-inflated negative binomial. Fit of the four models were compared by examining the Akaike Information Criteria, Bayesian Information Criteria and log-likelihood values. Zero-inflated models were also tested for overdispersion using the scaled Pearson chi-square. Sensitivity analyses explored whether results varied by child BMI classification, with separate models run for children classified as healthy weight (< 85th percentile; n = 78) and those classified as overweight or obese (≥ 85th percentile; n = 50). Child BMI z-score was omitted from the stratified model, all other hypothesized predictors included in the original model were preserved. Unadjusted and adjusted odds ratios (OR), incident risk ratios, 95% confidence intervals (95% CI) and *p*-values are reported. Statistical analyses were conducted using the GENMOD procedure in SAS Version 9.4.

## Results

Table 1 presents participants’ baseline characteristics. Most parents were female (98.4%) and Hispanic (97.6%). Families attended an average of three workshops (mean = 3.35, SD = 2.48), with nearly three fourths (71.1%) attending at least one. Table 2 shows the model fit characteristics between the four models that were computed to examine predictors of engagement. As shown, the zero-inflated Poisson showed better fit than the Poisson. There were very few differences between the zero-inflated Poisson and zero-inflated negative binomial models in terms of fit indices, but the lack of evidence of overdispersion (p > .05) indicated the zero-inflated Poisson model may be better suited for the data. Therefore, a zero-inflated Poisson (ZIP) model was used to examine predictors of parent engagement.

**Table 1:** Characteristics of Participating Parents and Children

	n (%) or Mean (SD)
*Parent (N = 128)*	
Age	35.34 (8.42)
Sex, Female	126 (98.4%)
Marital Status, Married	94 (73.4%)
Ethnicity, Hispanic	124 (97.6%)
Education, ≥High school diploma	77 (60.2%)
Employed	43 (33.6%)
Enrolled in public assistance	98 (76.6%)
Perceived relevance (Range: 0–5)	3.26 (1.12)
Readiness to change (Range: 0–5)	4.32 (0.55)
Perceived barriers (Range: 0–3)	0.46 (0.51)
Family Functioning (Range: 1–4)	3.59 (0.74)
Psychological Health Symptoms (Range: 0–12)	2.29 (2.95)
Perception of child weight, Underestimated	79 (61.7%)
Healthy weight (BMI < 25)	21 (16.4%)
*Child (N = 128)*	
Age	6.82 (2.91)
Sex, Female	64 (20.0%)
Healthy Weight (BMI percentile < 85%)	78 (60.9%)
Behavioral Health Issues (1+)	21 (16.4%)
Total family workshop attendance (Range: 0–6)	3.35 (2.48)
Degree of workshop attendance 0 workshops 1 + workshops	37 (28.9%) 91 (71.1%)

SD, Standard deviation; PHQ, Patient Health Questionnaire; BMI, body mass index

**Table 2:** Model Fit Characteristics

Model Type	Log-likelihood	AIC	BIC
Poisson	-408.32	844.645	884.464
Negative Binomial	-342.07	712.146	751.965
Zero-inflated Poisson	-239.903	535.806	615.444
Zero-inflated Negative Binomial	-242.071	542.14.473	624.624

AIC, Akaike’s Information Criteria; BIC, Bayesian Information Criteria

Unadjusted and adjusted regression results are shown in Table 3. The ZIP analysis yielded two models. The first model examined predictors of zero-values, or non-attendance in this case, via a logistic regression model (i.e., attendance at 0 workshops versus attendance at 1 + workshops). A second Poisson or count model examined predictors of degree of attendance. In unadjusted models, no variables were significantly predictive of attendance. In the adjusted zero-inflated model including all hypothesized predictors, only readiness to change predicted attendance. Specifically, there was an inverse relationship such that parents with a lower level of readiness to change had higher odds of attending no workshops (OR = 0.419, *p* < .05). In the Poisson model, only family functioning significantly predicted degree of attendance. Families with better family functioning attended more workshops (RR = 1.55, *p* < .01).

**Table 3:** Zero-inflated Poisson Models for Parent Workshop Attendance, Unadjusted and Adjusted (N = 128)

	Unadjusted Models^a^						Adjusted Model^b^							
	**Logistic**			**Poisson**			**Logistic**			**Poisson**				
	OR	95% CI	*p*	RR	95% CI	*p*	OR	95% CI	*p*	RR	95% CI	*p*		
*Parent*														
Age	0.98	− 0.06, 0.29	0.45	1.00	0.98, 1.01	0.92	0.96	-0.11, 0.03	0.22	0.99	0.98, 1.01	0.49		
Married	0.66	-1.26, 0.45	0.35	1.08	0.86, 1.37	0.48	0.67	-1.54, 0.73	0.49	1.08	0.84, 1.40	0.54		
Enrollment in public assistance	0.47	-1.61, 0.11	0.08	1.11	0.91, 1.34	0.29	0.58	-1.58, 0.73	0.31	1.08	0.87, 1.35	0.47		
Perceived Relevance	1.16	-0.20, 0.51	0.39	1.01	-0.07, 0.09	0.73	1.22	-0.43, 0.83	0.53	0.99	0.89, 1.10	0.86		
Readiness to Change	0.54	-1.33, 0.11	0.09	1.05	0.88, 1.24	0.57	**0.41**	**-1.70, -0.04**	**0.04**	1.07	0.90, 1.27	0.44		
Particpation Barriers	1.34	-0.44, 1.03	0.43	1.13	0.94, 1.35	0.18	1.38	-0.59, 1.24	0.49	1.11	0.90, 1.37	0.32		
Family Functioning	1.12	-0.48, 0.71	0.71	1.14	0.97, 1.33	0.10	2.33	-0.52, 2.21	0.22	**1.25**	**1.06, 1.48**	**0.009**		
Psychological health symptoms	1.10	-0.03, 0.22	0.13	1.00	0.97, 1.04	0.70	1.17	-0.07, 0.36	0.12	1.01	0.97, 1.06	0.56		
Underestimate Child Body Weight	0.97	-0.83, 0.78	0.95	1.01	0.83, 1.23	0.90	0.75	-1.30, 0.72	0.57	1.05	0.84, 1.29	0.68		
BMI	1.01	-0.05, 0.06	0.78	1.00	0.98, 1.01	0.99	0.97	-0.10, 0.05	0.49	1.00	0.99, 1.02	0.73		
*Child*														
Age	0.71	-0.22, 0.04	0.19	1.00	0.96, 1.03	0.097	0.94	-0.23, 0.13	0.58	0.99	0.96, 1.04	0.98		
BMI *Z-score*	1.25	-1.65, -0.64	0.15	1.01	0.93, 1.10	0.71	1.43	-0.09, 0.71	0.11	1.05	0.95, 1.16	0.32		
Behavioral Health Issues (1+)	2.82	-0.29, 2.37	0.13	0.89	0.71, 1.13	0.37	2.61	-0.58, 2.49	0.22	0.82	0.63, 1.07	0.16		

^a^Each cell represents a single model; ^b^ All variables included in the model

OR, Odds Ratio; 95%CI, 95% Confidence interval; RR, rate ratio; BMI, body mass index

Sensitivity analyses in which separate adjusted models including all hypothesized predictors were run for children classified as healthy weight (n = 78) and those classified as overweight or obese (n = 50) at baseline suggest that results may vary by child BMI classification (not shown but available upon request). Specifically, in the adjusted models among families with children with a healthy BMI, a similar pattern to the results of the full sample emerged, such that the relationship between readiness to change and non-attendance was significant (OR = 0.33, p < .05), and better family functioning predicted a higher degree of attendance (RR = 1.57, *p* < .01). By contrast, in models that included only families with children with overweight or obesity, no significant predictors of non-attendance or degree of attendance emerged.

## Discussion

Family-based programs have shown promise in the prevention and control of childhood obesity; however, their efficacy may be limited by low parent engagement [8]. This study fills several knowledge gaps regarding parent engagement, which has been limited by methodological constraints (e.g., retrospective designs) and largely consists of clinical treatment studies [12]. Notably, study results show different processes may affect engagement in different program phases (e.g., during recruitment versus during intervention) and engagement may differ by participant characteristics, such as participant’s baseline weight status. This finding indicates a need for retention strategies that are tailored to the unique needs of various participants and that can be adapted throughout the program. Similar conclusions have been drawn by others in the field [12, 39], such as LoBraico and colleagues (2021), who observed sociodemographic and psychosocial differences between children and families who ended their participation in a family-based health intervention early and those with sustained attendance [39].

Results indicate parents’ readiness to make changes, both in their own behaviors and in parenting strategies, were predictors of non-attendance at planned intervention activities. This finding is critical because readiness to change is potentially modifiable. For instance, one study found that parent’s readiness to make changes can be influenced by getting information from their provider [40]. Researchers and practitioners should consider tailoring their communication about childhood obesity interventions, as well as intervention content, based on family’s readiness to change. One potential approach is to incorporate brief motivational interviewing at the time of enrollment [41] as well as during the intervention itself, as was done in CA-CORD. Another approach is improved communication at baseline about the child’s BMI classification or health status, as numerous studies have shown both that parents are likely to underestimate their children’s’ weight and that those who do not perceive their child as overweight or obese have a lower readiness to change [26, 42]. By using appropriate, non-stigmatizing language to communicate weight-related health issues [43, 44], interventionists and/or providers could increase perceived need for intervention, and subsequently parents and children may be more likely to engage. However, as this finding was observed only among healthy weight children, more research is needed to determine if the same approaches would be effective among those classified as overweight or obese.

Family functioning emerged as a predictor of degree of attendance. These results align with other research showing a link between family functioning and engagement in childhood obesity programs [5], and to child weight status directly [45]. These results are consistent with a qualitative examination of parent engagement in this same group of parents [46]. In interviews with a sub-set of participants, those with higher levels of engagement frequently described how their participation and ability to make healthy weight-related changes at home were facilitated by support from family members, including the participating child. For instance, in that study, one participant described how her children’s enthusiasm for the program encouraged her engagement, “*They [the children] were always supporting me, because they are always the ones that were rushing me and asking me what day it was going to be, how many days were left, and things like that.”* [46] Clearly, family functioning and communication can affect childhood obesity program attendance, but there is still much to be learned regarding how best to involve parents, caretakers, siblings and other family members in obesity prevention and control efforts [21]. Considering that families often engage in weight-related behaviors together, it is important to understand how to leverage and address existing family dynamics when designing programs—even if the intervention requires attendance only from parents [47]. Ultimately, family systems theory should serve as the foundation of family-based childhood obesity prevention and control efforts, and practitioners should identify means of assessing and addressing family organization and communication around weight-related behaviors throughout the duration of the program [19, 21].

### Strengths and Limitations

Several factors related to the study population and design must be considered when interpreting the results. First, the relatively small, homogenous sample and low response rate among CA-CORD participants (29.8%) may limit generalizability. Notwithstanding, participants represented a predominately Hispanic/Latinx population with higher-than-average socioeconomic barriers to health, a group that has historically been underrepresented in research. Therefore, results provide valuable information about a unique population and on how to reach similarly high-risk groups. Second, this study used attendance to measure engagement. Attendance is an important objective measure of engagement, but future studies may benefit from also incorporating assessments of active participation in intervention activities.

Third, despite the robust, theoretically-driven list of predictors assessed and prospective design, the model did not account for program experiences that could affect engagement. Research suggests satisfaction with the program and/or program leaders can positively impact engagement [14], and therefore it may be beneficial to assess it at multiple points (e.g., after each workshop). Similarly, the design did not allow for observation of changes that may occur during program participation that could affect engagement, such as positive changes in weight, behaviors, or family functioning. For instance, participants who experience weight loss or successfully change certain behaviors shortly after participation begins may be more likely to remain engaged [48]. Thus, in addition to using a prospective design to examine predictors of engagement, future studies could benefit from repeated measures designs that collect behavioral or health data during the program, and not just before and after participation. Fourth and finally, this study did not overtly assess external social, environmental, structural factors [49] that could further contextualize the parents’ experiences that could impact engagement. While indicators of income and transportation access were assessed, future studies may benefit from exploring the impact of other factors, such as neighborhood safety, housing status, and/or working conditions, on parent engagement in family-based programs.

## Conclusion

As rates of childhood obesity in the U.S. rise, so does the need for effective interventions. This study provides important insight into factors that could improve parent engagement in family-based childhood obesity prevention and control programs, which could potentially improve program outcomes. Of note is the identification of modifiable psychosocial predictors. Researchers and practitioners should make concerted efforts to incorporate strategies to maximize engagement throughout the program, such as motivational interviewing during enrollment to increase readiness to change. Further, the role of family functioning should not be overlooked when designing and implementing family-based programs. Using family systems theory as a foundation, future programs should assess and possibly address family communication and family member roles throughout the program to boost engagement. Finally, these results indicate different engagement strategies may be needed for families seeking to prevent overweight or obesity versus those with children who are already overweight.

## Data Availability

The datasets used and/or analysed during the current study are available from the corresponding author on reasonable request.

## List of abbreviations

CHW: community health worker
CA-CORD: Imperial County, California, Childhood Obesity Research Demonstration study
BMI: Body mass index
ZIP: zero-inflated Poisson
OR: Odds Ratio
95%CI: 95% Confidence interval
RR: rate ratio
AIC: Akaike’s Information Criteria
BIC: Bayesian Information Criteria
